# Sub-population analysis based on temporal features of high content images

**DOI:** 10.1186/1471-2105-10-S15-S4

**Published:** 2009-12-03

**Authors:** Merlin Veronika, James Evans, Paul Matsudaira, Roy Welsch, Jagath Rajapakse

**Affiliations:** 1 Computation and Systems Biology, Singapore-MIT Alliance, Nanyang Technological University, Singapore 637460; 2 Bioinformatics Research Centre, Nanyang Technological University, Singapore 637553; 3 Department of Biological Science, National University of Singapore, Singapore 117543; 4 Whitehead Institute for Biomedical Research, Cambridge, Massachusetts 02139, USA; 5 Sloan School of Management, Massachusetts Institute of Technology, Cambridge, Massachusetts 02142, USA

## Abstract

**Background:**

High content screening techniques are increasingly used to understand the regulation and progression of cell motility. The demand of new platforms, coupled with availability of terabytes of data has challenged the traditional technique of identifying cell populations by manual methods and resulted in development of high-dimensional analytical methods.

**Results:**

In this paper, we present sub-populations analysis of cells at the tissue level by using dynamic features of the cells. We used active contour without edges for segmentation of cells, which preserves the cell morphology, and autoregressive modeling to model cell trajectories. The sub-populations were obtained by clustering static, dynamic and a combination of both features. We were able to identify three unique sub-populations in combined clustering.

**Conclusion:**

We report a novel method to identify sub-populations using kinetic features and demonstrate that these features improve sub-population analysis at the tissue level. These advances will facilitate the application of high content screening data analysis to new and complex biological problems.

## Background

Cell motility is an essential component of normal development, inflammation, tissue repair, angiogenesis, and tumor invasion [1]. After conception, selected cells of the developing mammalian zygote invade the uterine wall to establish the placenta, while the intricately programmed migration of other cells within the embryo shapes the complex form of the emerging organism. Fetal nervous system presents us with an example of large scale migration of immature neuroblasts from their place of origin to other locations in order to make right connections [2]. Studies about cell migration pattern have been commonly performed at the population level. Although such analysis gives a general idea of what is happening, they are void of what happens at the sub-population level [3]. A common example is cancer, where variation in cancer cells leads to therapeutic treatments that target a particular subset of the population [4,5]. Therefore, for studies aiming at potential therapeutic treatment, it is important to focus on sub-populations that can be treated to prevent recurrence and metastasis.

Several studies have developed techniques to mine and infer biological knowledge from high-content screening (HCS) data. Perlman *et al *employed the Kolomogorov Smirnov statistic for high throughput cytological profiling. Their study reflected more on biological mechanism rather than chemical implications [6]. Tanaka *et al *used Principal Component Analysis to identify small molecule kinase inhibitors in morphological based screens and neural networks were used by Bakal *et al *to perform a genetic screen to identify genes controlling different aspects of cell morphology [7,8]. Support vector machines (SVM) and factor analysis were employed to identify compound activity [9,10]. These studies have demonstrated the need for simultaneous analysis of features, but the computational and graphical difficulties remain an unsolved challenge for data analysis [11]. The few existing analytical methods used to validate HCS data across a series of experiments require extensive operator interaction and, more importantly lack statistical rigour, resulting in underutilization of information available from the powerful HCS technology. As HCS applications become increasingly complex, so does the composition of the cell populations as well as the underlying covariance structure of the cellular data.

In order to overcome these bottlenecks, we propose to develop a sub-population analysis technique based on kinetic features. Our technique is semi-supervised and aims to find the correlation between cell motility (kinetic features) and cell morphology (static features) and infer biological mechanisms underlying cell motility of tissues under different conditions. This will facilitate studies on molecular signalling pathways involved in cell motility. In this manuscript, we illustrate our sub-population analysis by applying it to a population of IC 21 macrophage cells. Our method consists of several phases: using active contours without edges [12] for segmentation, autoregressive models [13,14] for modeling cellular trajectories and k-means [15] clustering to classify the cells.

## Results and discussion

### Dataset

The cells used in this experiment were mouse macrophage cell lines IC-21 (American Type Culture Collection (ATCC) TIB-186). The cell line was cultured in 85% RPMI-1640 medium, 15% FBS, and antibiotics (50 IU penicillin and 50 *μ*g/ml streptomycin). Cell culture and maintenance techniques were performed as described by ATCC. Cells are plated overnight to allow them to attach onto 96-well plate. The media is aspirated and incubated in 1:1000 dilution CMFDA. The media is aspirated again and new media containing 200 *μ*M Trolox is added. Cells were imaged over a period of 75 minutes at every 15 minute interval using Cellomics KineticScan giving a total of six snapshots. The cells do not undergo mitotic division during this time due to absence of activating factors. Four fields per well are imaged and each field contained approximately 125 cells. Each image is 8 bit grayscale (0-255) of size 1024 × 1024 pixels.

### Cell segmentation

**Figure 1:**
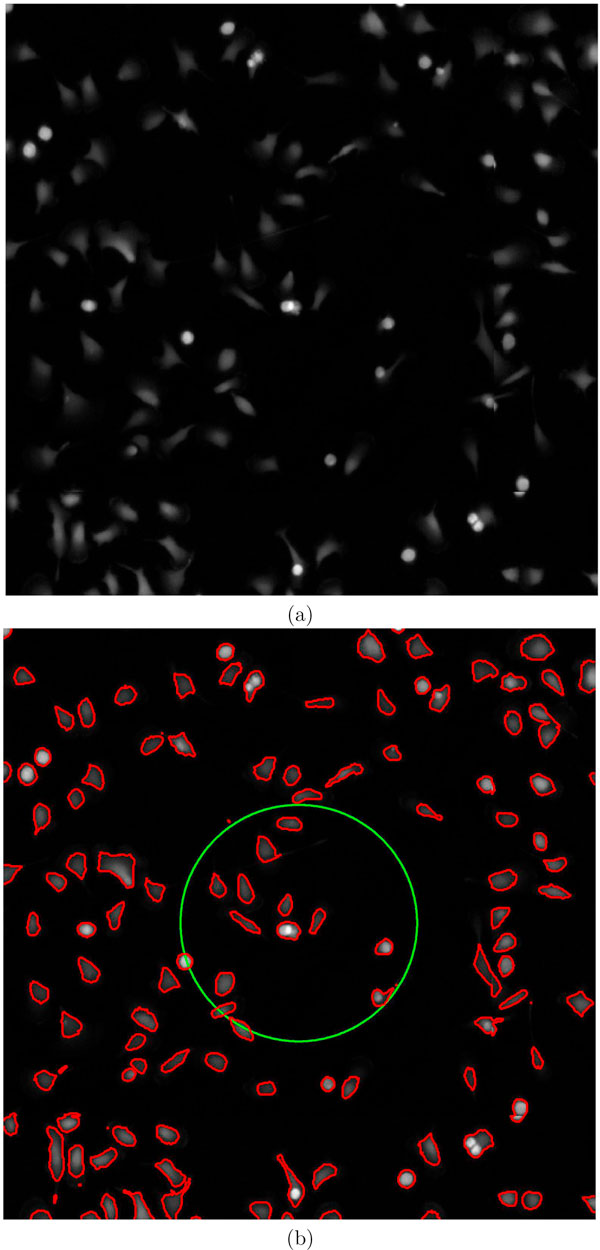
**Segmentation by level set method**. (a) Snapshot of the image data used for analysis (b) Initial (green) and final (red) contour representing segmented cells.

**Figure 2:**
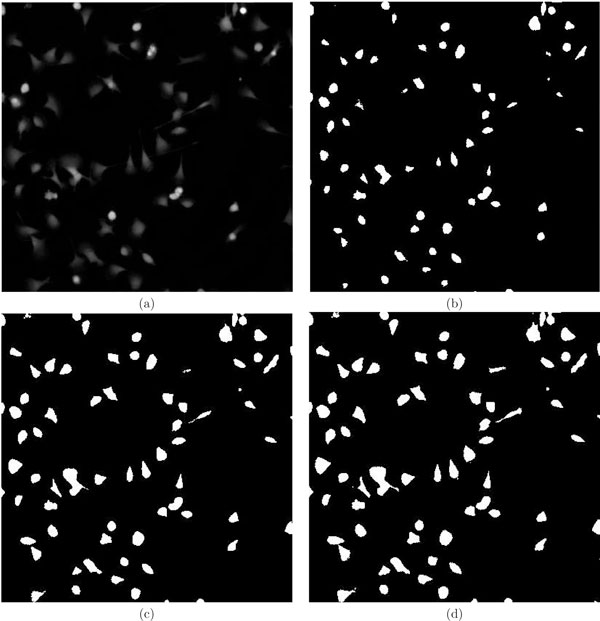
**Comparison of level set segmentation with Otsu's and fuzzy c-means method**. (a) Snapshot of unprocessed image. (b) Otsu's method. (c) Fuzzy c-means and (d) Level set segmentation.

Macrophage cells are identified by level sets, preserving the topology of the cells that is vital for extracting the static parameters. The results are furnished in Fig 1. Almost all the cells were properly identified by this method. Fig 1(a) shows a snapshot of unprocessed image. Fig 1(b) shows the segmented cells (enclosed in red contour). The CPU time for this method is 17.4 mins compared to classical Otsu method which took 1 *s *and fuzzy c-means clustering (50 *s*) on a 1.86 Ghz and 1 GB RAM desktop computer [16,17]. Segmentation is the first step in any image analysis pipeline and extraction of static features depend on how well shape features are conserved during segmentation. This method was compared with classical Otsu and fuzzy c-means, which resulted in severe loss of morphological information (Fig 2).

### Static features

Ensemble of basic and Zernike shape features were measured using MatLab's Image Processing toolbox (ver 6.1 R2008a). Zernike features describe more intuitive aspects of the cell and are calculated using an orthogonal basis set, the Zernike polynomials, which are defined over the unit circle. The amplitude of these complex-valued moments were used as features in subsequent analysis [18]. For the current analysis, Zernike polynomials from order 0 to 9 were calculated, giving in total 7 basic and 30 Zernike measurements. While there is no limit to the order which can be calculated, we found that this number of features was sufficient.

### Kinetic features

**Figure 3:**
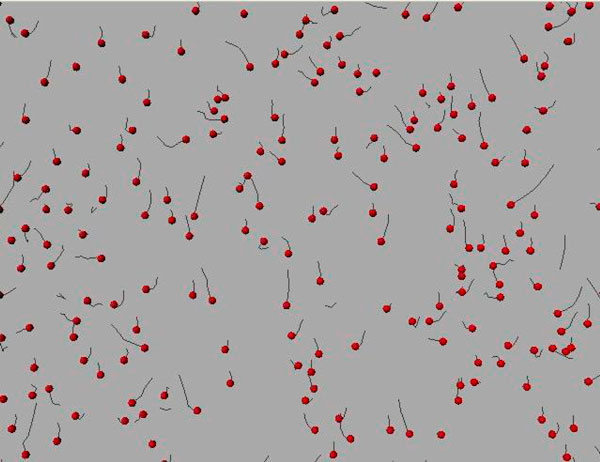
**Tracks of cells identified by AR Model**. Red spots represent initial position of the cell and dragon tail in black represent the trajectories.

Snapshot of tracks identified by auto-regressive models (AR) are shown in Fig 3. As we can observe, the movement of cells are anisotrophic. Features like (i) speed of cell, (ii) chemotactic index, (iii) length of the trajectory, and (iv) total displacement of the cell were measured using the method described above.

### Sub-population analysis

In K-means clustering, each cell is treated as an object having a location in multidimensional space. It finds a partition in which the cells in one cluster are close to each other and far off from cells belonging to other clusters. Eventually, total sum of the distances levels out even if data is partitioned into multiple clusters. The point of inflection indicates optimal cluster number.

**Figure 4:**
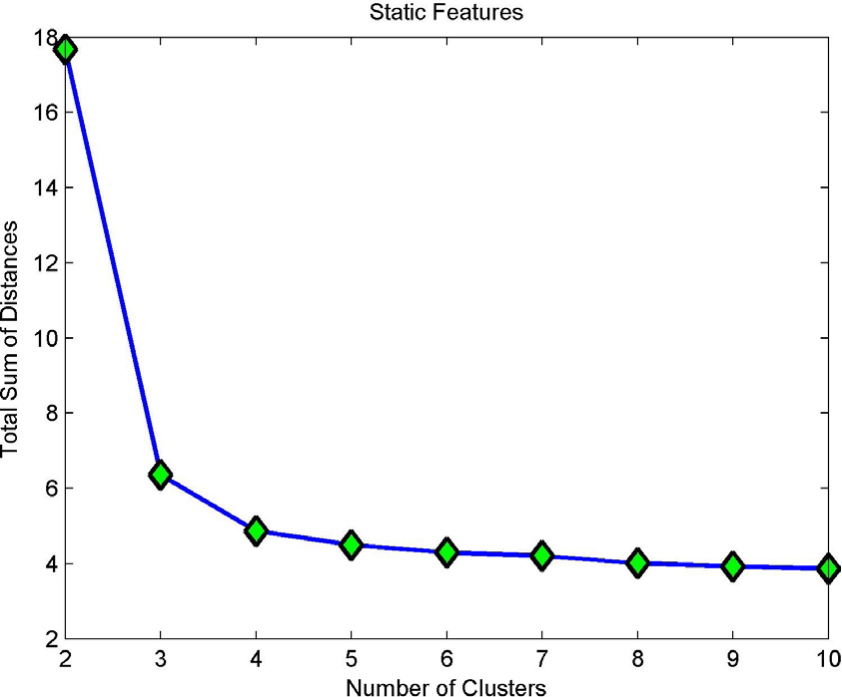
**Optimal clusters from static features**. For static features, the point of inflection is in cluster 3.

**Figure 5:**
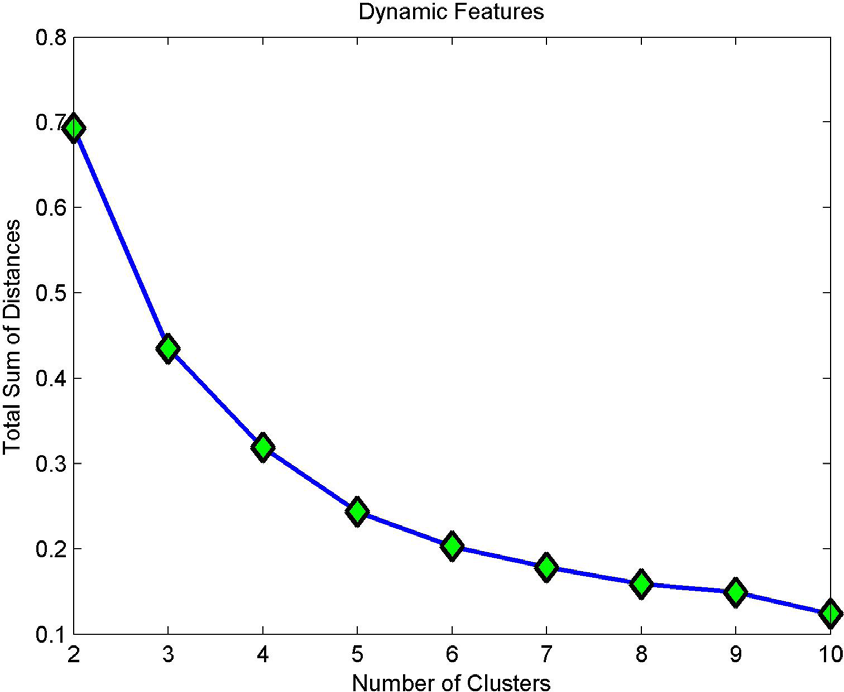
**Optimal clusters from dynamic features**. For dynamic features, the point of inflection is in cluster 4.

**Figure 6:**
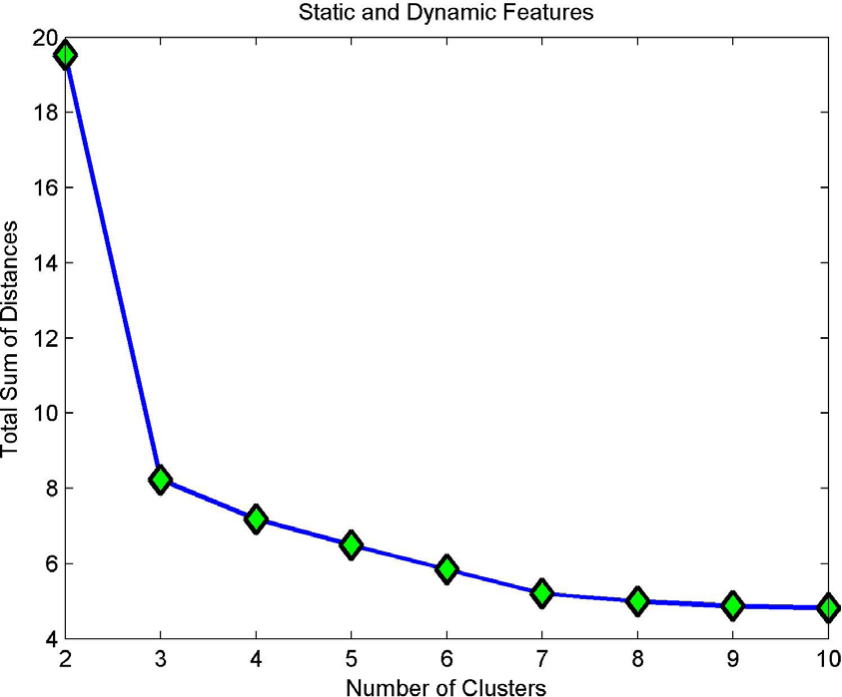
**Optimal clusters from static and dynamic features**. Adding dynamic features has increased the resolution of cluster size bringing the optimal cluster size to 3.

**Figure 7:**
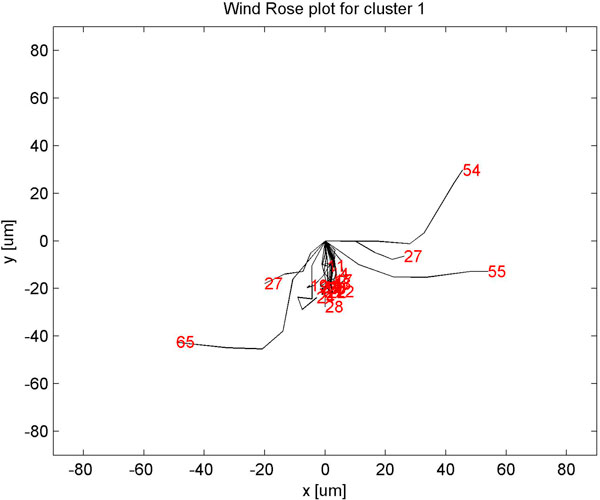
**Wind rose plot for cluster 1**. Wind rose plot for cluster 1. The numbers in red indicate total path length in *μ*

**Figure 8:**
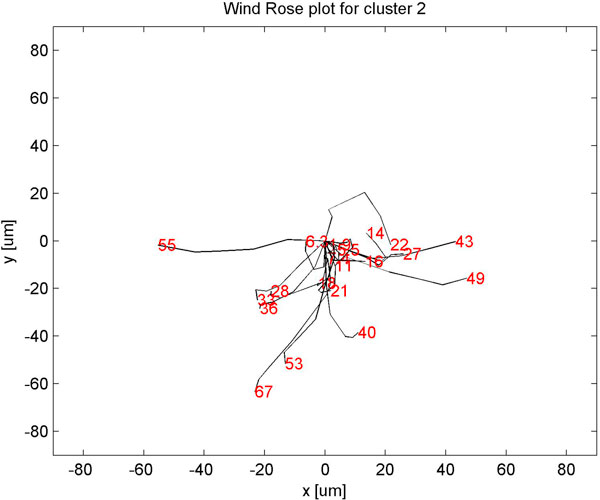
**Wind rose plot for cluster 2**. Wind rose plot for cluster 2. The numbers in red indicate total path length in *μ*

**Figure 9:**
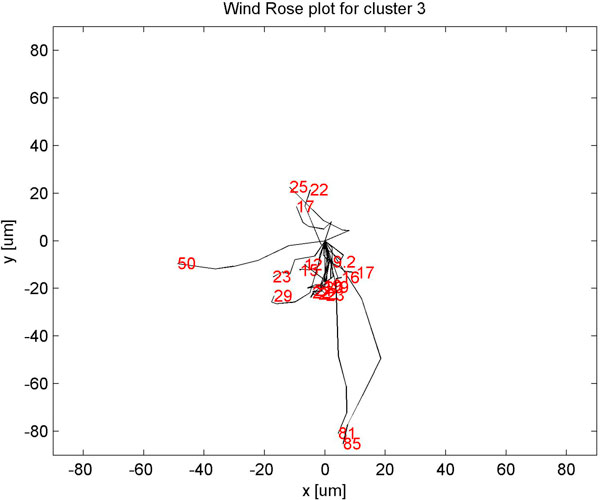
**Wind rose plot for cluster 3**. Wind rose plot for cluster 3. The numbers in red indicate total path length in *μ*

Clustering static features showed three clusters (Fig 4) with minimum homogeneity and maximum separation, whereas clustering with dynamic features showed four clusters with maximum homogeneity and minimum separation (Fig 5). Only when dynamic features were combined with static, did we get better resolution clusters which are homogeneous and well separated as shown in Table 1. Fig 6 shows optimal cluster number for combined static and dynamic features clustering. The three clusters of cells identified from the data can be described as follows: (i) Cluster 1: Compact cells which move or slide against the 2D surface steadily. This population formed the bulk and they had fan a shaped spread suggestive of active mobility. (ii) Cluster 2: Cells with much bigger area and perimeter. The speed of migration is the maximum among the three clusters showing directional bias. (iii) Cluster 3: Bigger cells which move very slowly and do not translate much. This is evident from low path displacement measure. This cluster showed clear directional bias and pattern of movement and is quite different from the previous clusters. A wind rose plot for top 20 cells representing each cluster shows the trend in directionality and variation in total displacement. A wind rose plot for cluster 1 is shown in (Fig 7), cluster 2 (Fig 8), cluster 3 (Fig 9). The features along with the mean values for each cluster are shown in Table 2.

**Table 1:** Validity indices for clustering based on static, dynamic and combination

Indices	Static	Dynamic	Both
Homogeneity Index	1.5825	0.3377	1.4810
Separation Index	1.1988	0.2924	0.9646
Weighted inter-to-intra cluster ratio	0.4981	0.4640	0.7831

**Table 2:** Features of individual clusters

Features	Cluster 1	Cluster 2	Cluster 3
Area (*μ*^2^)	224.41	447.94	222.22
Eccentricity	0.7400	0.7406	0.7582
Orientation (degree)	-49.69	-11.47	49.88
Solidity	0.9182	0.8826	0.9191
Extent	0.6386	0.6117	0.6443
Perimeter (*μ*)	69.70	106.02	69.92
Formfactor	0.7639	0.6624	0.7590
Zernike_0_0	0.5646	0.5508	0.5416
Zernike_1_1	0.2130	0.2115	0.2029
Zernike_2_0	0.1297	0.1236	0.1264
Zernike_2_2	0.0686	0.0693	0.0688
Zernike_3_1	0.1115	0.1075	0.1078
Zernike_3_3	0.0577	0.0523	0.0561
Zernike_4_0	0.0322	0.0244	0.0351
Zernike_4_2	0.0256	0.0260	0.0286
Zernike_4_4	0.0252	0.0194	0.0240
Zernike_5_1	0.0396	0.0346	0.0411
Zernike_5_3	0.0389	0.0345	0.0381
Zernike_5_5	0.0234	0.0189	0.0235
Zernike_6_0	0.0160	0.0119	0.0124
Zernike_6_2	0.0153	0.0125	0.0159
Zernike_6_4	0.0164	0.0111	0.0154
Zernike_6_6	0.0120	0.0088	0.0139
Zernike_7_1	0.0238	0.0187	0.0238
Zernike_7_3	0.0231	0.0190	0.0227
Zernike_7_5	0.0222	0.0173	0.0223
Zernike_7_7	0.0141	0.0096	0.0134
Zernike_8_0	0.0147	0.0075	0.0144
Zernike_8_2	0.0097	0.0074	0.0118
Zernike_8_4	0.0135	0.0076	0.0117
Zernike_8_6	0.0094	0.0066	0.0112
Zernike_8_8	0.0117	0.0069	0.0103
Zernike_9_1	0.0161	0.0124	0.0162
Zernike_9_3	0.0159	0.0116	0.0161
Zernike_9_5	0.0151	0.0101	0.0140
Zernike_9_7	0.0158	0.0106	0.0149
Zernike_9_9	0.0103	0.0066	0.0099
Speed (*μ*/h)	31.011	30.839	31.136
Chemotactic Index	0.8377	0.7816	0.8217
Path Length (*μ*)	33.946	33.812	33.543
Path displacement(*μ*)	29.310	27.837	28.426

## Conclusion

We have demonstrated a novel exploratory method of identifying sub-populations by combining static and dynamic features, in particular how dynamic features improve sub-population analysis at the tissue level. This kind of analysis is important in transplantation studies because macrophages being the sentries of immune systems, accumulate at the site of transplantation and the number and type of inflammatory cells often are more related to the surgical trauma and material [19]. Static features like area, eccentricity and perimeter are indicators of the protrusive forces that steer the cell to move forward or retract and are the main indicators to study the impact of any perturbation on macrophage cells. This is further supported by kinetic features like speed, chemotactic index and path length. The changes in these features indicate the response of cells to stimulus which in turn depends on the milieu where the cell is positioned.

Since our approach is semi-supervised, no prior knowledge or little intervention is required. This study also showcases the importance of kinetic features needed to quantify sub-populations. Features such as area, eccentricity, perimeter, speed, chemotactic index, path length/displacement capture all the essential information required to characterize any group of cells. There is much to be done to understand the intricacies of the features in play during cell motility. We outlined a framework that can be easily reproduced by using common data mining techniques (k-means) to gain insight into morphological and motility features obtained from HCS image data.

Our future work will focus on bridging cell morphology with cell motility to understand the underlying biological mechanisms. Not many studies have been dedicated to understanding the spatio-temporal relation at the sub-population level especially by making use of kinetic features. Understanding how these sub-populations change with drug treatment will give insight to how any morphology is related to one another and with motility factors. In particular, this framework will help in associating cell morphology with function which might be useful in detecting aberrant cancer cells or the differentiating potential of stem cells.

## Methods

Let us denote a set of coordinates of the pixel site in the image by Ω ⊂ ℝ^2 ^and the image *μ *is denoted as a function *f *: Ω → *Q*, where *Q *is the range of all possible intensities. Intensity at each pixel *x*, where *x *∈ Ω is given by *f*(*x*).

### Segmentation

Segmentation of tissue images involves separating a tissue image into individual cells. It is done by identifying regions with common properties or identifying contours which delineate regions. So, a natural way to segment such regions is through thresholding the intensity. This method is optimal for thresholding large objects and those with fairly distinct classes, but does not work well with small objects with blurry edges [16]. Active Contour Models (ACM), first introduced by Kass *et al*, represent an intelligent way of detecting boundary edges by considering boundaries as inherently connected and smooth structures [20]. An energy term is associated with the contour and is designed to be inversely proportional to the contour's smoothness and fit to the desired image features. Certain forces can be designed (or derived from energy terms) in a way that the resulting contour deformations will reduce the contour's energy. Because of the way the contours slither while minimizing their energy, ACM are also called snakes. The contour is said to possess an energy given by the sum of the three energy terms: internal, external and constraints. The energy terms are defined in such a way that the final position of the contour will have a minimum energy and therefore the problem of detecting objects reduces to an energy minimization problem. A caveat for active contours is that cells are under segmented when the border between the clustered cells are much brighter than the border between cell and background.

Because classical snakes and active contour models rely on the edge-function, depending on the image gradient, to stop the curve evolution, these models can detect objects only with edges defined by the gradient [21]. In practice, the discrete gradients are bounded and then the stopping function is never zero on the edges, and the curve may pass through the boundary. If the image is very noisy, then the isotropic smoothing gaussian has to be strong, which will smooth the edges too. Tony and Vese proposed a different active contour model, without a stopping edge-function, i.e, a model which is not based on the gradient of the image *f*(*x*) for the stopping process [12]. The stopping term is based on Mumford-Shah segmentation techniques [22]. The energy function of the active contour based on this function is given by:

where *ϕ *is the level set function defined on Ω whose zero level set {(*x*) ∈ Ω|*ϕ*(*x*) = 0} defines the segmentation such that *ϕ *> 0 is inside the cell and *ϕ *< 0 is outside the cell. *c*_*I *_and *c*_*O *_are mean intensities of pixels inside and outside the zero level set. *H *and *δ *are the Heaviside and Dirac functions. *α*, *λ*_*I *_and *λ*_*O *_are fixed positive parameters.

The minimization of image energy is achieved by evolving the level set for time *t*, starting from an initiation *ϕ *(*t *= 0, *x*) according to,

where ▽. is the mean curvature of the level set, generating a regulating force which smoothens the contours. The two forces expand or shrink the contour towards the actual boundary of the cells. We segmented the cells using the above method.

### Modeling cell trajectories

Among the different models developed for describing a stochastic process, auto-regressive model (AR) is perhaps the most popular [13,14]. The practical utility of an AR model becomes compelling when the stochastic process is non-stationary especially biological cell movement which sustains spatio-temporal patterns. An AR model computes the position of a cell *o *at time *t *based on the previous positions by,

where *o*(*t*) is the centroid of the cell at time *t*, *β*_0 _is a constant mostly ignored for simplicity purposes, *β*_*τ *_are autoregressive parameters, and *ε*(*t*) is the noise level at time *t *included to cover the possible cell positions.

### Quantifying cell motility

Eukaryotic cell migration in isotropic environments can be described as a persistent random walk. Over short time periods, cells follow a relatively straight path, showing persistence of movement. If long time intervals are used to observe the cell position, however, cell movement appears similar to Brownian motion with frequent direction changes. If a cell is executing a random walk, its expected distance (or displacement) <*d *> of its centroid from its original position varies with time according to the formula.

where <*d*^2 ^> denotes the mean square displacement of the cell, *γ *is the random motility coefficient (formally equivalent to a diffusion coefficient), and *m *is a constant giving the dimensionality of the random walk. According to the above formula, the average distance travelled by a cell is proportional to the square root of the elapsed time. Although they cover short distances rapidly, cells performing random walks travel long distances much more slowly.

At least two parameters are needed to describe a persistent random walk [23]. The first characteristic of cell movement is the persistence time *ρ *which is the measure of the average time between significant direction changes. The second motility parameter is the cell speed *ν *that is intuitively defined as the displacement of the cell centroid per unit time. If the speed is computed in this fashion, care must be taken to use time intervals small enough so that cells move in a constant direction. The persistence time *ρ *and cell speed *ν *can also be rigorously defined using mathematical analysis. Starting from different assumptions about the details of cell paths, [24,25] developed the following mathematical model to describe persistent random walks:

For long times (*t *>> *ρ*), the above formula reduces to the much simpler expression:

The persistent random walk analysis is applicable only when cell movement takes place in an isotropic environment. Modifications are necessary to analyze biased cell movement (e.g., in the presence of a chemoattractant) or to check whether cell locomotion has a preferred direction. One such approach is based on the stochastic concept of Markov chains.

The method proposed by Dickinson and Tranquillo uses a generalized non-linear regression algorithm wherein the cell tracks are assumed to consist of a sequence of cell positions associated with a series of increasing time points differing by a constant time increment [26]. If *o*(*t*) represents the centroid of the cell at time *t*, then the squared displacement *d*^2^(*τ*) of the cell over time interval *τ*, from *o*(*t*) to *o*(*t *+ *τ*), is:

then, *d*^2^(*τ*) is considered a random variable with expected value *η*_*τ *_≡ <*d*^2^(*τ*) > = <*d*^2^(*t*) >, where *η*_*τ *_is the theoretical mean-squared displacement over *τ*.

To obtain the measured mean squared displacement of any cell, several squared displacements over the cell track should be averaged. Two obvious and commonly used sampling methods, overlapping and nonoverlapping can be used. The total number of samples available from a single track is maximized by averaging squared displacements from overlapping time intervals. However, it is not statistically independent. An alternate method is to average only nonoverlapping intervals. Speed and persistence were calculated by fitting mean square displacement to a persistent walk model. Since the distance travelled in the given time is known, speed was calculated directly and persistence by fitting the model.

### Cell clustering

The K-means algorithm is one of the most popular iterative descent clustering methods and is used for clustering features gathered from HCS analysis [3,27]. It is intended for situations in which all variables are of the quantitative type. Each cluster is defined by a cluster centroid which is the mean of the features of the cluster population. The optimal number of clusters from K means were determined by three different indices; weighted inter to intra cluster ratio (Wint), homogeneity index and separation index. In Wint, squared euclidean distances, normalised for distinct variances of different populations are computed for pairs of cells within and across clusters. By weighting these average distances we restrict outlier cells from influencing the number of populations. Homogeneity index reflects the compactness of the cluster and is the average distance between the cells in the cluster and its respective centroid. Separation index is a measure of between-cluster variance defined by weighted average distance between clusters. Decreasing homogeneity index and increasing separation index suggest better clusters.

## Competing interests

The authors declare that they have no competing interests.

## Authors' contributions

MV conceived, designed and wrote the paper. JE and PM provided the cellular images and designed analysis, RW conceived and contributed statistical analysis part, JR conceived, designed and helped write paper. All authors read and approved the final manuscript.

## Note

Other papers from the meeting have been published as part of *BMC Genomics* Volume 10 Supplement 3, 2009: Eighth International Conference on Bioinformatics (InCoB2009): Computational Biology, available online at http://www.biomedcentral.com/1471-2164/10?issue=S3.
